# Actinomycosis

**Published:** 1895-08

**Authors:** W. C. Barth


### ACTINOMYCOSIS.[1]

BY W. C. BARTH, V.S.

THE disease of cattle known scientifically as actinomycosis, and popularly as "lumpy jaw," has attracted much attention in the United States on account of its great prevalence and dangerously contagious character. Actinomycosis is a pathological condition produced by the irritation of the ray fungus (actinomyces). Although this disease has existed for a long period in certain animals in this country, it is only a few years since its true nature was understood.

There can scarcely be any doubt as to the etiology of this disease. Actinomyces are constantly found in new formations of a special kind, and through their irritating and disintegrating influence not only produce these formations, but, sooner or later, set up certain destructive processes in the tissues in which they may locate, unless they lose their power or are removed. Therefore, they must be designated "infection tumors," and actinomycosis an infectious disease, as the tumors offer certain distinctive characters, and all tumors possessing these characters contain the fungi.

Externally these growths may be large or small and present various appearances, but they are generally round, lobular, or fungiform in shape, smooth on the surface, and soft in consistency. In color they may be grayish white or of light yellow tint. In the soft variety are generally seen a multitude of small very yellow nodules, whose presence is rarely a diagnostic feature. On section the typical character of the actinomykoma is best displayed. Imbedded in the fibrous stroma of the growth are noticed the various-sized nodules, more or less numerous, small and isolated, or in rounded masses the size of a hazelnut or walnut, gray or yellow in color, of a cheesy softness, and in the

[1] Read before the June meeting of the Missouri Valley Veterinary Association.

very smallest of them are a number of minute particles or centres, sulphur-yellow in tint, which are the clusters of the actinomyces. If one of the cheesy masses is submitted to pressure, bodies resembling millet-seeds are slightly separated from the matter around them. The majority of these are soft, something like tallow; others impregnated with lime-salts. These peculiar particles must always be considered diagnostic of the disease, even without the aid of the microscope. When the nodules are removed from the connective-tissue stroma this is found to be cavernous in its structure, another characteristic feature of the tumor.

I have already alluded to the pathology of the nodules, and to the manner in which, and channel by which the fungus invades the tissues. It is, however, extremely probable that it enters, in the form of spores, through a wound, abrasion, fissure, or even by means of the delicate mucous follicles of the membrane lining the lips, mouth, pharynx, and nostrils; in fact, any part of the digestive or respiratory canal. This injury may be inflicted in many ways, and very likely by the food upon which the animals most liable to disease are fed. The malady is most frequent after cattle have been fed on straw, barley, and chaff, and this may not only injure the mouth, but serve also as the vehicle of transmission of the fungus. Straw being so often mouldy and infected by vegetable parasites of various kinds, these spores may find their way into the œsophagus, stomach, and intestines or into the bronchi and lungs, and there fructify.

That this disease is transmissible from one animal to another has been experimentally demonstrated.

Prognosis must depend not only upon the locality or anatomical seat of the disease, but also upon the extent to which it has developed itself. When an important organ is involved, and that extensively, or when the disease is but slightly advanced, but is beyond reach, then the prognosis must be unfavorable. When it is accessible and has not caused serious alteration, and when it can be removed within a short time, then it may be pronounced favorable.

Sometimes spontaneous recovery takes place, probably owing to the fungus losing its vitality through diminished nutritive supply from retraction of the connective-tissue stroma, becoming encapsulated in lime-salts.

Cases that are treated with good results are the different lesions where the tumor is not connected with the bone, but lying loose in the connective tissue under the skin, or when the inferior maxilla does not exceed the size of a goose-egg, and the superior maxilla the size of a walnut. But in all cases the iodide of potassium treatment will avert further development of the disease.

Post-mortem examination of three hundred animals were made, and great care was exercised that accurate information might be gained. Incidentally, however, a number of interesting facts were observed. Fortunately many of the animals were still in that stage of the disease which permitted the accurate determination of the channels of infection. The autopsies in these cattle were limited to the head, thorax, abdomen, and glands.

In order to present these cases more intelligibly I shall mention in the order named: Actinomycosis of the glands of the head. In the head the disease was restricted mainly to the post-pharyngeal, submaxillary, lymphatic, and parotid lymph-glands, which were diseased in forty-one cases. In ten cases in which the only discoverable lesions were in the retro-pharyngeal glands, the actinomyces must have been carried from the nose or mucous membrane of the mouth or pharynx into the glands. The disease in the thoracic organs is of considerable interest to us, for it confirms the statement that a good many cases of bovine actinomycosis are of pulmonary origin. This means that the actinomyces are taken up and carried by currents of air as dust into the ramification of the tubes.

Of three hundred and sixteen cases, seventy-four were situated in the chest, the disease involving the bronchial glands and lung tissue, including air-tubes. In fifty-two cases the superior maxilla and in sixty-four the inferior maxilla were diseased, with no evidence of the disease elsewhere. In twenty cases the inferior maxilla gland and lungs were also diseased. In thirteen cases that were of the superior maxilla and lungs varied to the extent they were involved. In forty-one cases that had only superficial actinomycotic tumors, all glands in the head and other parts of the body were in perfect health, and in these cases the lungs were diseased, some more infiltrated than others. There were eighty-two animals that had only superficial actinomycotic tumors; all other parts of carcass in perfect health.

Disorganization of bone, apparently of a colloid nature, is found. The swollen bony structure is arranged in bands and layers, the space between being filled with gelatinous, reddish or somewhat white, substance and pockets containing yellowish pus of rather shiny character, and several cavities filled with soft red tissue, interrupted by the characteristic small yellow spots containing actinomyces. The maxillary sinuses are very often filled with granulation-tissue, and the bordering bones are rarefied. Often actinomycotic new-formations extend into the nasal fossæ, almost extending into the nostrils.

By this brief statement we can see that the disease is found mostly in the inferior maxilla. This form is not the most serious. But where we have the glands and the lungs involved this form is the most dangerous, but the most favorable to treat.

